# Signature Patterns for Top-Down and Bottom-Up Information Processing via Cross-Frequency Coupling in Macaque Auditory Cortex

**DOI:** 10.1523/ENEURO.0467-18.2019

**Published:** 2019-04-30

**Authors:** Christian D. Márton, Makoto Fukushima, Corrie R. Camalier, Simon R. Schultz, Bruno B. Averbeck

**Affiliations:** 1 Centre for Neurotechnology, and Department of Bioengineering, Imperial College London, London SW7 2AZ, United Kingdom; 2 Section on Learning and Decision Making, Laboratory of Neuropsychology, National Institute of Mental Health/National Institutes of Health, Bethesda, Maryland 20892; 3 RIKEN Center for Brain Science Institute, Saitama 351-0106, Japan; 4 Consumer Neuroscience, The Nielsen Company, Tokyo 107-0052, Japan

**Keywords:** auditory cortex, cross-frequency coupling, information processing, neural coding, predictive coding, top down

## Abstract

Predictive coding is a theoretical framework that provides a functional interpretation of top-down and bottom-up interactions in sensory processing. The theory suggests there are differences in message passing up versus down the cortical hierarchy. These differences result from the linear feedforward of prediction errors, and the nonlinear feedback of predictions. This implies that cross-frequency interactions should predominate top-down. But it remains unknown whether these differences are expressed in cross-frequency interactions in the brain. Here we examined bidirectional cross-frequency coupling across four sectors of the auditory hierarchy in the macaque. We computed two measures of cross-frequency coupling, phase–amplitude coupling (PAC) and amplitude–amplitude coupling (AAC). Our findings revealed distinct patterns for bottom-up and top-down information processing among cross-frequency interactions. Both top-down and bottom-up interactions made prominent use of low frequencies: low-to-low-frequency (theta, alpha, beta) and low-frequency-to-high- gamma couplings were predominant top-down, while low-frequency-to-low-gamma couplings were predominant bottom-up. These patterns were largely preserved across coupling types (PAC and AAC) and across stimulus types (natural and synthetic auditory stimuli), suggesting that they are a general feature of information processing in auditory cortex. Our findings suggest the modulatory effect of low frequencies on gamma-rhythms in distant regions is important for bidirectional information transfer. The finding of low-frequency-to-low-gamma interactions in the bottom-up direction suggest that nonlinearities may also play a role in feedforward message passing. Altogether, the patterns of cross-frequency interaction we observed across the auditory hierarchy are largely consistent with the predictive coding framework.

## Significance Statement

The brain consists of highly interconnected cortical areas, yet the patterns in directional cortical communication are not fully understood, in particular with regard to interactions between different signal components across frequencies. We used a novel, convenient, and computationally advantageous Granger-causal framework to examine bidirectional cross-frequency interactions across four sectors of the auditory cortical hierarchy in macaques. Our findings reveal that cross-frequency interactions are predominant in the top-down direction, with important implications for theories of information processing in the brain such as predictive coding. Our findings thereby extend the view of cross-frequency interactions in auditory cortex, suggesting that they also play a prominent role in top-down processing.

## Introduction

One fundamental yet poorly understood component of cortical computation is the presence of widespread reciprocal cortical connections (Salin and Bullier, 1995; Kveraga et al., 2007; Romanski and Averbeck, 2009; Scott et al., 2017). Moreover, most previous work has focused on the visual pathways. It remains unknown whether there are fundamental differences between top-down (TD) and bottom-up (BU) information processing along the auditory pathway. Cross-regional top-down effects in particular have remained unexplored, as most work has focused on bottom-up (Giraud and Poeppel, 2012; Hyafil et al., 2015a) or intraregional effects (Lakatos et al., 2016). In the present study, we examined bidirectional information processing across the macaque auditory hierarchy through an analysis of cross-frequency coupling (CFC). We computed two types of CFC measures, amplitude–amplitude coupling (AAC) and phase–amplitude coupling (PAC), for both natural and synthetic stimuli.

Cross-frequency coupling refers to the correlation between the phase or amplitude component of one frequency and the amplitude component of other frequencies. This differs from linear analyses like coherence, which only examine coupling between signals at a single frequency. CFC, in contrast, considers interactions across the frequency spectrum. CFC may occur in several forms (Jirsa and Mueller, 2013) and has been observed during various tasks across several species including humans, macaques, and rodents. PAC, in particular, has received widespread attention. Coupling of low-frequency (e.g., theta, alpha) phase to high-frequency (e.g., gamma) amplitude has been associated with various behavioral mechanisms including learning, memory, and attention (Schack et al., 2002; Canolty et al., 2006 ; Jensen and Colgin, 2007; Cohen, 2008; Tort et al., 2008, 2009; Canolty and Knight, 2010; Kendrick et al., 2011; Doesburg et al., 2012 ; Colgin, 2015 ; Hyafil et al., 2015b). The reason for interest in PAC is that it may offer a mechanistic account of information coordination across neural populations and timescales. The phase of low-frequency oscillations could modulate the amplitude of high-frequency oscillations, putatively allowing control of spiking activity (Ray and Maunsell, 2011; Buzsáki et al., 2012). AAC has also been shown to correlate with behavior (Canolty and Knight, 2010), but has been less explored mechanistically. Here we obtained estimates of both PAC and AAC strength as measures of cross-regional information processing in the bottom-up and top-down directions, allowing the two measures to be contrasted and compared in terms of informational content.

One prevalent theory of information processing in the brain that separates bottom-up and top-down components is predictive coding. In this (Bayesian) view of the brain, expectations are formed about incoming sensory information. These top-down predictions (expectations) are compared with bottom-up sensory information (outcomes) and representations are then potentially updated based on a prediction error (surprise), which is the difference between sensory inputs and their expectations. Neural implementations of predictive coding suggest that predictions are formed in higher-order areas through a (linear) accumulation of prediction errors. The accumulation of prediction errors in higher-order areas results in the attenuation of high frequencies; thus, feedforward connections can be cast as a low-pass or Bayesian filter. Conversely, prediction errors arising in lower-order areas are a nonlinear function of predictions. This means high frequencies can be created in lower-order areas due to the inherent nonlinearity. Hence, predictive coding implies that lower-order areas (where prediction errors arise) should display relatively higher frequencies (e.g., gamma range) than higher-order areas (where predictions are formed; Friston, 2008; Bastos et al., 2012). Several studies have supported this view (Bastos et al., 2012, 2015; Fontolan et al., 2014; Michalareas et al., 2016), though not without exceptions.

It remains unknown whether this prediction is expressed in cross-frequency interactions in the brain. Here we probe this prediction by examining differences in cross-frequency coupling strength in the bottom-up and top-down directions across the frequency spectrum. Based on the predictive coding framework (Friston, 2008; Bastos et al., 2012), we would expect there to be an asymmetry between bottom-up and top-down interactions. This asymmetry arises due to the linear effect of prediction errors on predictions in the bottom-up direction and the nonlinear effect of predictions on prediction errors in the top-down direction. Thus, we would expect nonlinear, cross -frequency interactions to predominate in the top-down direction.

Previous work has shown differences in how auditory cortical areas process natural and synthetic sounds (Fukushima et al., 2014). It is possible that these differences translate into systematic differences in CFC patterns. If the revealed CFC pattern is a more general hallmark of interareal communication, though, it should not be specific to stimulus type: natural and synthetic stimuli should show similar overall coupling patterns.

Our work revealed signature patterns of top-down and bottom-up processing in cross-regional PACs and AACs. These patterns were conserved across coupling types and across natural and synthetic stimuli. We found that low-to-low-frequency (theta, alpha, beta) and low-frequency-to-high-gamma interactions were predominant top-down, while low-frequency-to-low*-*gamma couplings were predominant bottom-up. Our findings suggest that the modulation of gamma frequency power by low-frequency rhythms plays a role in bidirectional information transfer across the auditory hierarchy. The finding of predominant cross-frequency interactions in the top-down direction across the auditory hierarchy largely agrees with the predictive coding framework.

## Materials and Methods

### Subjects

Three adult male rhesus monkeys (*Macaca mulatta*) weighing 5.5–10 kg were used for recordings. All procedures and animal care were conducted in accordance with the Institute of Laboratory Animal Resources *Guide for the Care and Use of Laboratory Animals*. All experimental procedures were approved by the National Institute of Mental Health Animal Care and Use Committee.

### Stimuli

The stimuli used for the main experiment included 20 conspecific monkey vocalizations (VOCs) and two sets of 20 synthetic stimuli each [envelope-preserved sound (EPS); and spectrum-preserved sound (SPS)] derived from the original VOC stimuli. The VOC stimulus set consisted of 20 macaque vocalizations used in previous studies (Kikuchi et al., 2010; Fukushima et al., 2014).

To obtain EPS from VOC stimuli, the envelope of a particular vocalization was estimated based on the amplitude component of the Hilbert transform of the original stimulus. The amplitude envelope was then multiplied by broadband white noise to create the EPS stimulus. Thus, all 20 EPS stimuli exhibited flat spectral content; these stimuli could not be discriminated based on spectral features, while the temporal envelopes (and thus the durations) of the original vocalizations were preserved.

SPS stimuli were obtained by first generating broadband white noise with a duration of 500 ms and computing its Fourier transform. The amplitude of the SPS stimulus in the Fourier domain was then replaced by the average amplitude of the corresponding VOC stimulus before transforming back to the time domain by computing the inverse Fourier transform. This resulted in a sound wave form that preserved the average spectrum of the original vocalization, while exhibiting a flat temporal envelope, random phase, with a duration of 500 ms. A 2 ms cosine rise/fall was then imposed on the stimulus to avoid abrupt onset/offset effects. Hence, all 20 SPS stimuli exhibited nearly identical, flat temporal envelopes; these stimuli could not be discriminated using temporal features, while the average spectral power of the original vocalizations was preserved.

A total of 60 different stimuli were presented in pseudorandom order, with an interstimulus interval of 3 s. Each stimulus was presented 60 times. The sound pressure levels of the stimuli measured by a sound level meter ranged from 65 to 72 dB at a location close to the animal's ear of the animal. Stimulus duration ranged from ∼0.15 to 1 s (Fukushima et al., 2014).

### Recordings

Custom-designed micro-electrocorticography (*µ*ECoG) arrays (NeuroNexus) were used to record field potentials from macaque auditory cortex. Arrays were machine fabricated on a very thin polyimide film (20 *µ*m), with each array featuring 32 recording sites, 50 *µ*m in diameter each, on a 4 × 8 grid with 1 mm spacing (i.e., 3 × 7 mm rectangular grid). Two animals, monkeys B and K, were both implanted with arrays in the left hemisphere, while one animal, monkey M, was implanted with arrays in the right hemisphere. Three of the arrays in each monkey were placed on top of supratemporal plane (STP) in a caudorostrally oriented row.

To implant the arrays, we removed a frontotemporal bone flap extending from the orbit ventrally toward the temporal pole and caudally behind the auditory meatus and then opened the dura to expose the lateral sulcus. The most caudal of the three ECoG arrays on the STP was placed first and aimed at area A1 by positioning it just caudal to an (imaginary) extension of the central sulcus and in close proximity to a small bump on the STP, both being markers of the approximate location of the A1. Each successively more rostral array was then placed immediately adjacent to the previous array to minimize interarray gaps. The arrays on the lateral surface of the STG were placed last. The probe connector attached to each array was temporarily attached with cyanoacrylate glue or Vetbond to the skull immediately above the cranial opening. Ceramic screws together with bone cement were used to fix the connectors to the skull. The skin was closed in anatomic layers. Postsurgical analgesics were provided as necessary, in consultation with the National Institute of Mental Health veterinarian.

### Stimulus presentation and recording parameters

The monkey was placed in a sound-attenuating booth for the experiment (Biocoustics Instruments). The sound stimuli were presented while the monkey sat in a primate chair and listened passively with its head fixed. Auditory evoked potentials from the 128 channels of the ECoG array were bandpassed between 2 and 500 Hz, digitally sampled at 1500 Hz, and stored on hard-disk drives by a PZ2–128 preamplifier and the RZ2 base station (Tucker-Davis Technologies).

### Data preprocessing

Data analysis was performed using MATLAB R2017a (MathWorks) software. Since there was little significant auditory evoked power >250 Hz, recordings were low-pass filtered and resampled at 500 Hz to enhance calculation speed and reduce memory requirements. The signal was bandfiltered at 60 Hz with a narrow-band filter to eliminate the possible presence of line noise; a sixth-order Butterworth filter was chosen to achieve a sharp drop-off in the stop-band, thus minimizing the effect of the filter on neighboring frequency bands.

**Figure 1.**
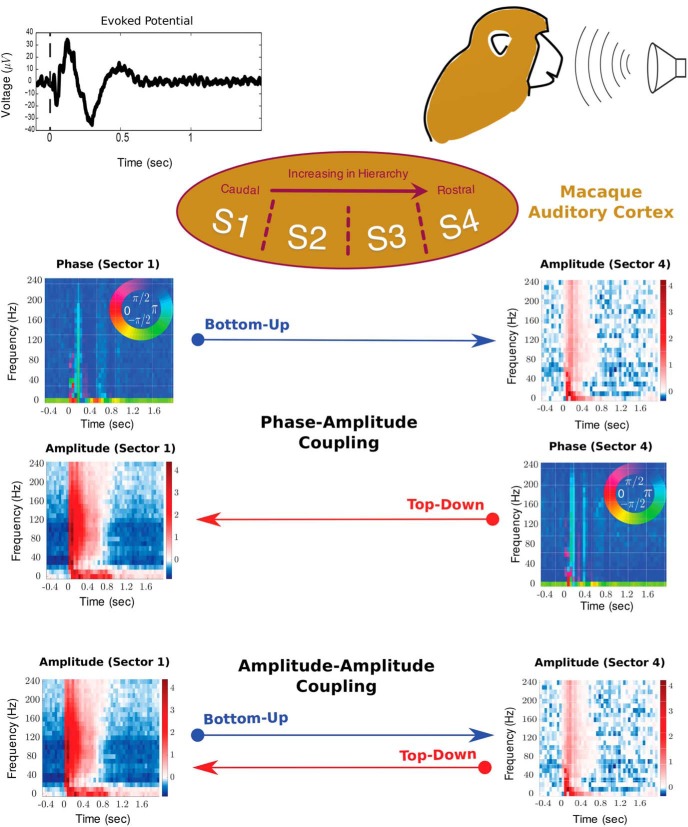
Coupling analysis overview: recordings were made from four *µ*ECoG arrays spanning auditory cortex while monkeys listened to 1 of 20 natural VOCs, 20 synthetic EPSs, or 20 synthetic SPSs. Electrodes were partitioned into four sectors along the caudorostral axis (S1, A1/ML; S2, R/AL; S3, RTL; S4, RTp). We decomposed the signal into its time–frequency representation, and obtained amplitude and phase components for each sector. Plots of signal amplitude (in dB) are normalized to the baseline activity before stimulus onset. We investigated two types of coupling across sectors: amplitude–amplitude and phase–amplitude coupling. We computed both types of coupling in the bottom-up and top-down directions. Top-down coupling was defined as coupling in which the source electrode came from a sector of higher order than the target electrode, and vice versa for bottom-up coupling.

The 96 sites on the STP were grouped based on the characteristic frequency maps obtained from the high-gamma power of the evoked response to a set of pure-tone stimuli. The change in frequency tuning along the STP reverses across areal boundaries, and these reversals were therefore used to identify the areal boundaries. This resulted in a grouping of electrodes into four sectors (Fig. 1), which were estimated to correspond to the following subdivisions at a caudorostral level within macaque auditory cortex: sector (Sec) 1, A1/ML (primary auditory cortex/middle lateral belt); Sec 2, R (rostral core region of the auditory cortex)/AL (anterior lateral belt region of the auditory cortex); Sec 3, RTL (lateral rostrotemporal belt region of the auditory cortex); and Sec 4, RTp (rostrotemporal pole area). The recorded signal from each site partitioned into sectors 1–4 was rereferenced by subtracting the average of all sites within a particular sector (Kellis et al., 2010; Fontolan et al., 2014).

We defined the following terminology to refer to particular frequency spaces throughout this article: delta range, 0–2.5 Hz; theta range, 2.5–7.5 Hz; alpha range, 7.5–12.5 Hz; low beta range, 12.5–22.5 Hz; high beta range, 22.5–42.5 Hz; low gamma range, 42.5–67.5 Hz; and high gamma range, *>*67.5 Hz. The delta frequency band (0–2.5 Hz) was not included in the analysis since the preamplifier used during the recording sessions does not allow for recording frequency components <2 Hz and the power in this band was expectedly low.

### Phase-amplitude (PAC) and amplitude-amplitude (AAC) cross-frequency coupling calculation

#### Frequency transformation

The field potential data was first transformed into frequency space by computing the fast Fourier transform (FFT) of the signal in every channel using a 200 ms Hanning window, stepped by 200 ms. Thus, we obtained a frequency representation of the data at a 5 Hz bandwidth ranging from 2.5 to 132.5 Hz, which satisfies the Nyquist criterion. We obtained the phase and amplitude information in a given source and target region for every channel and normalized the amplitude information in each frequency band by the corresponding power in that frequency band, averaged across the entire experiment, so as to account for decreasing power with frequency (Extended Data Fig. 2-1).

**Figure 2.**
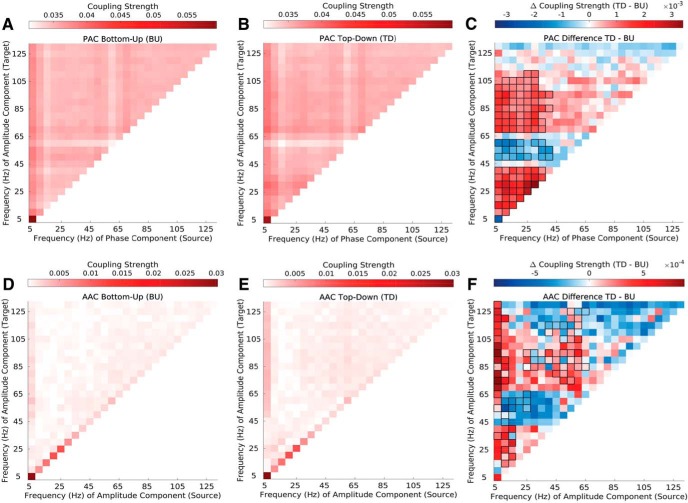
Top-down vs bottom-up phase–amplitude and amplitude–amplitude coupling in natural vocalizations (VOC). ***A***, ***B***, Phase-amplitude coupling (PAC) strength in the top-down (***A***) and bottom-up (***B***) directions. Depicted are canonical correlation-derived coupling coefficients (see Materials and Methods). ***C***, Difference in top-down and bottom-up in PAC strength. Significant differences are enclosed by black rectangles (*p* ≤ 0.01, cluster-corrected). ***D***, ***E***, Amplitude-amplitude coupling (AAC) strength in the top-down (***D***) and bottom-up (***E***) directions. Depicted are canonical correlation-derived coupling coefficients. ***F***, Difference between top-down and bottom-up in AAC strength. Significant differences are marked by black outlines (*p* ≤ 0.01, cluster corrected). Results are depicted averaged across all channels, cross-regional pairs, and animals. See Extended Data Figure 2-1, Extended Data Figure 2-2, and Extended Data Figure 2-3.

10.1523/ENEURO.0467-18.2019.f2-1 Extended Data Figure 2-1 NEED LEGEND. Download Figure 2-1, EPS file.

#### Obtaining CFCs using canonical correlation

**Figure 3.**
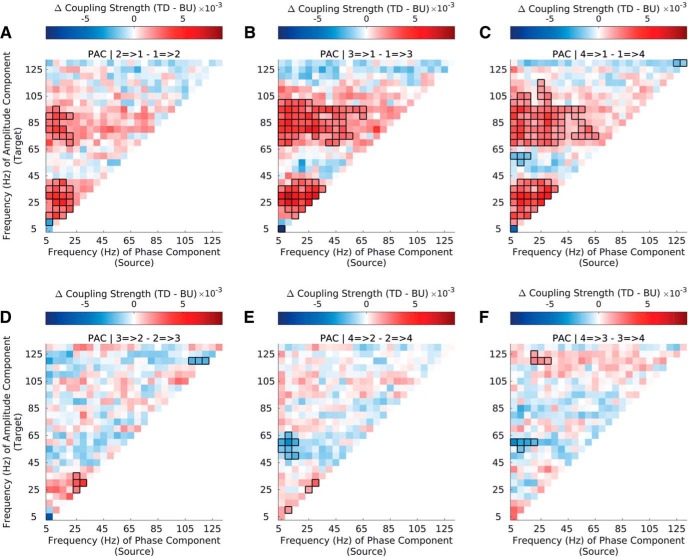
Top-down vs bottom-up PAC strength across the auditory hierarchy in natural stimuli. ***A–F***, Difference in top-down and bottom-up PAC strength for CFC between sectors 1 (A1/ML) and 2 (R/AL; ***A***), sectors 1 (A1/ML) and 3 (RTL; ***B***), sectors 1 (A1/ML) and 4 (RTp; ***C***), sectors 2 and 3 (***D***), sectors 2 and 4 (***E***), and sectors 3 and 4 (***F***; for sector definitions, see Data preprocessing, in Materials and Methods ). Interactions between sector 1 (A1/ML) and higher-order sectors show strong asymmetries in bottom-up and top-down coupling strength across the frequency spectrum; interactions among higher-order sectors (S2–S4) show less widespread asymmetries. Significant differences are marked by black outlines (*p ≤* 0.01, cluster corrected). Results are depicted averaged across all channels and animals. See Extended Data Figure 3-1.

We computed two types of cross-frequency coupling, PAC and AAC. Couplings were calculated for every channel pair across the four sectors (S1–S4), resulting in six possible cross-sector pairs (S1–S2, S1–S3, S1–S4, S2–S3, S2–S4, and S3–S4). We obtained estimates of coupling strength in two directions, bottom-up and top-down. In the former case, the source component was derived from a recording site in a sector lower in the auditory hierarchy than the target component (referred to as “bottom-up coupling”), while in the latter case the source component came from a sector higher in the auditory hierarchy than the target component (referred to as “top-down coupling”; Fig. 1). Ultimately, we were interested in examining the difference in coupling strength in the top-down and bottom-up directions across the auditory hierarchy. Results are presented collapsed across all cross-sector pairs (Fig. 2), as well as separately for every cross-sector pair (Fig. 3), averaged across all channel pairs and across the three animals in both cases.

10.1523/ENEURO.0467-18.2019.f2-2 Extended Data Figure 2-2 NEED LEGEND. Download Figure 2-2, EPS file.

10.1523/ENEURO.0467-18.2019.f2-3 Extended Data Figure 2-3 NEED LEGEND. Download Figure 2-3, EPS file.

10.1523/ENEURO.0467-18.2019.f3-1 Extended Data Figure 3-1 NEED LEGEND. Download Figure 3-1, EPS file.

We used canonical correlation analysis (CCA) to compute both types of coupling. The advantage of CCA is that it computes coupling among multivariate observations in the source and target regions. This framework, therefore, allows CFC values to be computed across the entire frequency range simultaneously. Furthermore, some approaches to calculating AAC and PAC first carry out dimensionality reduction on the source and target region signals separately, and then calculate correlations in the low-dimensional space. If the variance preserving dimensions in the source and target space are not the dimensions that maximize correlations between the source and target space, interactions may be missed. CCA on the other hand finds the low-dimensional representations that maximize coupling between the source (*X*) and target (*Y*) space. In CCA, one is seeking projections (linear combinations) *U* = *X A* and *V* = *Y B*, such that the correlation corr(*U*, *V)* among these derived directions is maximal (Durstewitz, 2017).

We conducted our analyses using a Granger causal framework. Therefore, we first conducted CCA between amplitude in the target region and time-lagged versions of the amplitude also within the target region, obtaining an estimate of the coupling strength Y^ that could be inferred purely from coupling of the signal at the target electrode onto itself. For computational reasons, we used two time lags to estimate the target signal (we found that using one time lag did not affect our results). More explicitly, we transformed the current and lagged signal into the frequency domain as described above and computed the canonical correlation between the lagged components and the current signal in the target. We used the first 10 canonical values only to obtain Y^ (results did not differ from those obtained from including more entries). We then obtained the residual $\overset{˜}{Y}$ from this analysis. Subsequent analysis was performed using the residuals $\overset{˜}{Y}$ from this regression as the signal for the target region.

We then computed PAC and AAC cross-frequency couplings across the four sectors (Fig. 1) by regressing the phase or amplitude component of the signal from the source sector, respectively, on the amplitude component of the signal in the target sector for every channel. More specifically, we calculated instantaneous coupling in 200 ms windows between the source and the target region for each electrode pair. PAC and AAC were calculated in the CCA framework across all time windows and trials together, simultaneously across all frequency bands. In the case of PAC, *X_n×d_*_1_ contained the sine and cosine transformed phase component of the signal, while *Y_n×d_*_2_ contained the squared and log-transformed amplitude component of the signal, with *n* = 10,800 (1200 trials × 9 windows) observations for VOC and SPS stimuli and *n* = 10,773 observations (1197 trials × 9 windows) for EPS stimuli, *d*2 = 25, and *d*1 = 25 or *d*1 = 25 × 2 for PAC and AAC, respectively (Eq. 1).

After obtaining the canonical coefficient matrices *B_d_*_2_*_×d_* and *A_d_*_1_*_×d_* within the CCA framework (Eq. 1), one can obtain the coupling matrix *P_d_*_2_*_×d_*_1_ by multiplying the *B* and *A* matrices with the canonical values *S* of the correlation matrix (Eq. 2). We used the first 10 directions (*d* = 10) that captured most of the interaction; results did not differ from those obtained from including all entries. For AAC, the final cross-frequency coupling matrix is *P* . For PAC, one combines the sine and cosine components of *P* by computing their Euclidian norm, yielding the final coupling matrix $P_{d2\;x\;d1}^{*}$ with *d*2 = *d*1:

(1) $$U_{n\times d}=X_{n\times d1}\;A_{d1\times d},\;V_{n\times d}={\overset{˜}{Y}}_{n\times d2}\;B_{d2\times d},\underset{A,B}{\text{argmax}\left\{\mathrm{corr}\left(U,V\right)\right\}}\;$$

(2) $$P_{d2\times d1}^{}=\mathrm{BS}A^{T},\;P_{d2\times d1}^{*}=(\overset{\sqrt{P_{1}^{2}+P_{m/2}^{2}}}{\underset{\sqrt{P_{m/2}^{2}+P_{m}^{2}}}{\begin{array}{c} \cdots \end{array}}}).$$

#### Obtaining the difference between TD and BU coupling

We obtained AAC and PAC matrices in this manner for both the bottom-up and the top-down direction. In bottom-up coupling, the source component was derived from a recording site in a sector lower in hierarchy than the target component (referred to as “bottom-up coupling”), while in top-down coupling, the source component came from a sector higher in hierarchy than the target component (Fig. 1). We then obtained the difference Δ*P*_same stim_ between the top-down and bottom-up coupling matrices *P*_Top−Down_ and *P*_Bottom−Up_ (Eq. 3). This was done separately for each of the cross-sector pairs. Results are presented collapsed across all cross-sector pairs (Fig. 2), as well as separately for every cross-sector pair (Fig. 3), as follows:

(3) $$\Delta P=P_{\mathrm{Top}-\mathrm{Down}}-P_{\mathrm{Bottom}-\mathrm{Up}}.$$

#### Obtaining the difference in coupling strength between original (VOC) and synthetic stimuli (EPS/SPS)

**Figure 4.**
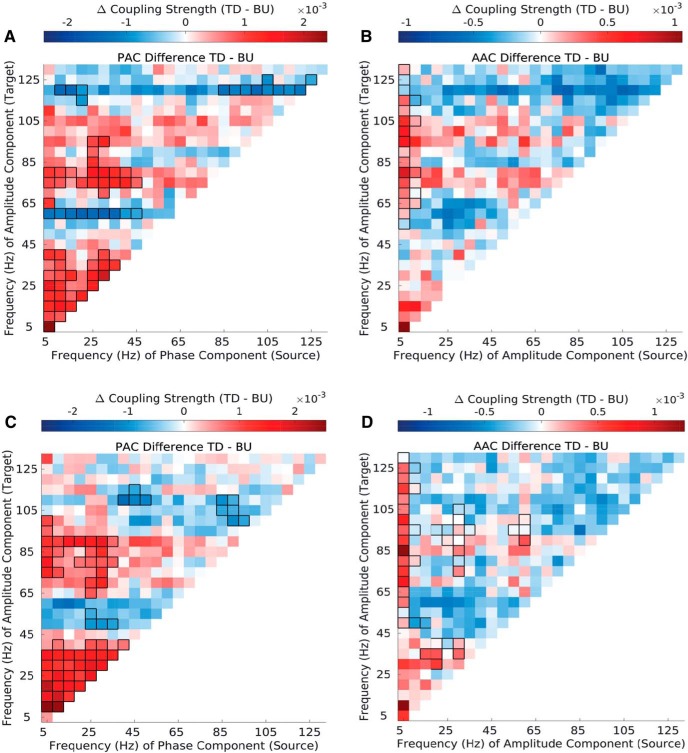
Top-down vs bottom-up phase–amplitude and amplitude–amplitude coupling in synthetic envelope-preserved stimuli (EPSs) and synthetic spectrum-preserved stimuli (SPSs) ***A***, Difference in top-down and bottom-up PAC strength in EPS stimuli. ***B***, Difference in top-down and bottom-up AAC strength in EPS stimuli. ***C***, Difference in top-down and bottom-up PAC strength in SPS stimuli. ***D***, Difference in top-down and bottom-up AAC strength in SPS stimuli. Significant differences are marked by black outlines (*p ≤* 0.01, cluster corrected). Results are depicted averaged across all channels, cross-regional pairs, and animals. See Extended Data Figure 4-1, Extended Data Figure 4-2, Extended Data Figure 4-3, Extended Data Figure 4-4, Extended Data Figure 4-5, and Extended Data Figure 4-6.

**Figure 5.**
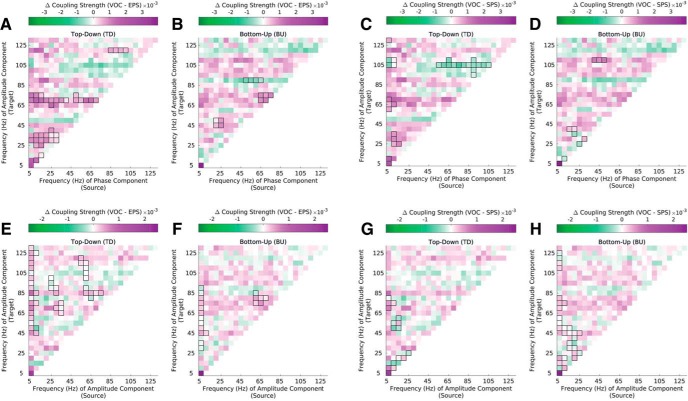
Phase-amplitude (PAC) and amplitude-amplitude (AAC) coupling strength in natural vocalizations (VOC) compared with synthetic envelope-preserved sounds (EPS) and synthetic spectrum-preserved sounds (SPS). ***A–D*** Difference in PAC strength between natural vocalizations and synthetic sounds. The difference between VOC and EPS stimuli (***A–B***) and between VOC and SPS (***C–D***) stimuli is depicted separately in the top-down and bottom-up direction. ***E–H*** Difference in AAC strength between natural vocalizations and synthetic sounds. The difference between VOC and EPS stimuli (***E–F***) and between VOC and SPS (***G–H***) stimuli is again depicted separately in the top-down and bottom-up direction. Significant differences are marked by black outlines (*p* ≤ 0.01, cluster corrected). Results are depicted averaged across all channels, cross-regional pairs, and animals. See Extended Data Figure 5-1.

To examine the difference in coupling strength between natural and synthetic stimulus types (Figs. 4, 5), we computed the difference between VOC and EPS stimuli (Fig. 4) and between VOC and SPS stimuli (Fig. 5) separately in the TD (Eq. 4) and BU (Eq. 5) directions.

(4) $$\Delta P_{\mathrm{VOC}\;\mathrm{vs}\;\mathrm{Synthetic}}^{\mathrm{TD}}=P_{\mathrm{VOC}}^{\mathrm{TD}}-P_{\mathrm{EPS}/\mathrm{SPS}}^{\mathrm{TD}}$$

(5) $$\Delta P_{\mathrm{VOC}\;\mathrm{vs}\;\mathrm{Synthetic}}^{\mathrm{BU}}=P_{\mathrm{VOC}}^{\mathrm{BU}}-P_{\mathrm{EPS}/\mathrm{SPS}}^{\mathrm{BU}}.$$

10.1523/ENEURO.0467-18.2019.f4-1 Extended Data Figure 4-1 NEED LEGEND. Download Figure 4-1, EPS file.

10.1523/ENEURO.0467-18.2019.f4-2 Extended Data Figure 4-2 NEED LEGEND. Download Figure 4-2, EPS file.

10.1523/ENEURO.0467-18.2019.f4-3 Extended Data Figure 4-3 NEED LEGEND. Download Figure 4-3, EPS file.

10.1523/ENEURO.0467-18.2019.f4-4 Extended Data Figure 4-4 NEED LEGEND. Download Figure 4-4, EPS file.

10.1523/ENEURO.0467-18.2019.f4-5 Extended Data Figure 4-5 NEED LEGEND. Download Figure 4-5, EPS file.

10.1523/ENEURO.0467-18.2019.f4-6 Extended Data Figure 4-6 NEED LEGEND. Download Figure 4-6, EPS file.

10.1523/ENEURO.0467-18.2019.f5-1 Extended Data Figure 5-1 NEED LEGEND. Download Figure 5-1, EPS file.

### Statistical analysis

Paired *t* tests were performed to establish statistical significance of the difference between top-down and bottom-up coupling (ΔP; Figs. 2, 3, 4), as well as the differences between original and synthetic stimuli ($\Delta P_{\mathrm{VOC}\;\mathrm{vs}\;\mathrm{Synthetic}}^{\mathrm{TD}}$, $\Delta P_{\mathrm{VOC}\;\mathrm{vs}\;\mathrm{Synthetic}}^{\mathrm{BU}}$; Fig. 5). This was done separately for each frequency pair, by testing whether the mean of the difference between top-down and bottom-up coupling (or the difference between original and synthetic stimuli in each direction) was significantly different from zero (null hypothesis). Lilliefors test for normality was conducted for each frequency pair to ensure that the normality assumption was satisfied, and histogram plots were inspected for select frequency pairs (Extended Data Fig. 2-2, Extended Data Fig. 2-3). More specifically, to assess overall differences in coupling strength between the two directions (Figs. 2, 4) and to assess differences in coupling strength between natural and synthetic stimuli (Fig. 5), paired *t* tests were conducted across all channel pairs, cross-regional pairs, and animals together (*df* = 9344). To assess coupling differences for each regional pair separately (Fig. 3), paired *t* tests were conducted separately for each regional pair, across all channel pairs and animals together (*df* = 1056).

Significant phase–amplitude and amplitude–amplitude coupling (highlighted by black outlines around the coupling values throughout the figures) was based on corrected *p* values using nonparametric permutation tests that generated null distributions of the maximum cluster size (Nichols and Holmes, 2002; Maris and Oostenveld, 2007). This procedure implicitly adjusts for searching over multiple frequencies. The null distribution was obtained by randomly reassigning electrodes to source or target region and recalculating differences in coupling strength for 100 repeats. Clusters of the 99th percentile (corresponding to *p ≤* 0.01) were considered significant and are reported throughout the manuscript (marked by black outlines).

## Results

We analyzed surface potentials recorded simultaneously from multiple cortical areas in the auditory cortex of three macaques while the animals listened to auditory stimuli (Fig. 1). These auditory stimuli consisted of 20 natural conspecific vocalizations, 20 EPSs, and 20 SPSs, which were derived from the original vocalizations (Fukushima et al., 2014). A previous study established the approximate rostrocaudal location of implanted *µ*ECoG arrays along the auditory hierarchy by the characteristic frequency of each electrode contact (Fukushima et al., 2012, 2014). This partitioned the recording sites into four sectors (S1–S4), putatively spanning the caudorostral levels from the core A1 (S1) to RTp (S4), including some of the surrounding belt areas (Fig. 1). We operationally defined feedforward to occur from earlier to later sectors (bottom-up direction), and feedback to occur from later to earlier sectors (top-down direction).

We wanted to examine cross-frequency interactions across the auditory hierarchy. To characterize phase–amplitude and amplitude–amplitude coupling, we first decomposed the signal into its spectral component using the short-time FFT and obtained phase and amplitude components (Fig. 1). We then used canonical correlation analysis (CCA) to parameterize the cross-frequency coupling (Materials and Methods). The fluctuations in amplitude in the target sector were predicted based on instantaneous amplitude fluctuations in the source sector within 200 ms windows. Furthermore, the target and source time series were adjusted to remove the effect of predicted fluctuations from the target region. In other words, by appealing to the notion of Granger causality, a significant mapping between the source and the target, which cannot be explained by the history of the target, is evidence of (cross-frequency) coupling.

We computed coupling strength across the frequency spectrum for each cross-regional pair, separately in the top-down and bottom-up direction (Fig. 1). Top-down coupling was defined as coupling in which the source electrode came from a sector of higher order than the target electrode, and vice versa for bottom-up coupling. For example, coupling between the phase (or amplitude) in S4 and the amplitude in S1 is defined as top-down, and coupling between the phase (or amplitude) in S1 and the amplitude in S4 is defined as bottom-up in the case of PAC (or AAC). This definition is in accordance with a previous approach (Fontolan et al., 2014).

### Distinct cross-frequency coupling signatures for bottom-up and top-down coupling, both involving low frequencies

We first examined PAC and AAC across the four sectors, in auditory evoked potentials to 20 conspecific VOCs. Both CFC measures peaked in the theta frequency range and showed relatively strong coupling across all the target amplitude frequencies (Fig. 2*A*,*B*,*D*,*E*). The field potential signal showed the highest power in the same frequency band decaying with the typical 1/*f* pattern thereafter (Extended Data Fig. 2-1), thus satisfying a prerequisite for the presence of physiologically meaningful CFC (Aru et al., 2015).

Among PACs, both top-down and bottom-up coupling were dominant between theta phase and broadband amplitude (Fig. 2*A*,*B*). Examining differences in coupling strength in the two directions more closely (Fig. 2*C*), PACs showed dominant top-down coupling in the low-frequency space (theta, alpha, and beta phase to theta, alpha, and beta amplitude), as well as between low-frequency phase and high-gamma (*>*67.5 Hz) amplitude (*p ≤* 0.01, cluster corrected). Bottom-up PAC was dominant between low-frequency phase (theta, alpha, beta) and low-gamma (47.5-62.5Hz) amplitude (*p ≤* 0.01, cluster corrected).

AACs showed a pattern similar to that of PACs in both directions, but coupling strength and differences in coupling strength were overall by an order of magnitude lower than those for PACs (Fig. 2*D*,*E*). AACs replicated the pattern observed in PACs, with dominant theta to broadband coupling in both directions. Additionally, and unlike in PACs, coupling strength among same-frequency couplings was pronounced in both directions with strongest coupling in the low-frequency range (Fig. 2*D*,*E*). Examining differences in coupling strength in the two directions more closely (Fig. 2*F*), AACs displayed a similar pattern to PACs but showed smaller significant clusters than PACs across the entire frequency spectrum.

### Cross-regional coupling mainly driven by interactions between A1 and higher-order sectors

To examine cross-regional patterns in coupling across the auditory hierarchy (Fig. 1) more closely, we analyzed differences between top-down and bottom-up coupling strength separately across the six cross-regional pairs (Fig. 3). This revealed that the overall coupling pattern (Fig. 2) was mostly driven by interactions between S1 (A1/ML) and S2-S4 (Fig. 3*A–C*), rather than by interactions among higher-order sectors (Fig. 3*D–F*).

More specifically, interactions between S1 (A1/ML) and S2 (R/AL)/ S3 (RTL)/ S4 (RTp) show dominant top-down coupling of low-frequency phase to low- and high-frequency amplitude (Fig. 3*A–C*), whereas these asymmetries are largely absent among interactions between higher order sectors (Fig. 3*D–F*). The interaction between S1 and S2 (Fig. 3*B*) shows overall less widespread top-down coupling strength compared with interactions between S1 and S3/S4. The full pattern (as manifested in Fig. 2) only gains full prominence among cross-regional pairs S1–S4 (Fig. 3*C*), which also includes predominant bottom-up coupling between low-frequency phase and low-gamma amplitude. Interactions between higher-order sectors (Fig. 3*D–F*) show fewer differences between top-down and bottom-up coupling, but they do show predominant bottom-up coupling between low-frequency phase and low-gamma amplitude (Fig. 3*E*,*F*). A similar trend was observed among AACs, although differences were overall smaller and less widespread compared with PACs (Extended Data Fig. 3-1).

### Top-down and bottom-up cross-frequency signatures generalize to synthetic stimuli

To examine whether the observed coupling patterns are a general feature of interareal communication, we also analyzed coupling strength in two types of synthetic stimuli derived from the natural vocalizations. EPSs were obtained by leaving the original temporal envelope of the vocalization intact but flattening its spectral content, while SPSs were obtained by leaving the spectral information of the original vocalization intact but flattening its temporal envelope (Fukushima et al., 2014; see Stimuli in Materials and Methods).

EPS and SPS stimuli showed similar bottom-up and top-down coupling patterns to natural stimuli, with pronounced theta-to-broadband coupling strength in both directions (Extended Data Fig. 4-1, Extended Data Fig. 4-4). Examining differences between top-down and bottom-up coupling strength in both types of synthetic stimuli (Fig. 4, Extended Data Fig. 4-1, Extended Data Fig. 4-4), we found that they reflected the same coupling asymmetries across the frequency spectrum as did natural stimuli (Fig. 2). This was true for PAC (Fig. 4*A*,*C*) and AAC (Fig. 4*B*,*D*) alike. More specifically, both types of synthetic stimuli showed dominant top-down coupling among low-to-low-frequency and low-to-high-gamma interactions, and dominant bottom-up coupling among low-to-low-gamma interactions, as did natural stimuli. Both types of synthetic stimuli also showed similar coupling asymmetries to natural stimuli when examining cross-regional pairs separately (Extended Data Fig. 4-2, Extended Data 4-3, Extended Data 4-5, Extended Data 4-6), with most of the pattern driven by interactions between A1 and higher-order sectors rather than interactions among higher-order sectors, as in natural vocalizations.

### Synthetic stimuli show overall lower bidirectional coupling than natural stimuli

To examine differences in coupling strength between natural and synthetic stimuli more closely, we compared coupling strength across stimuli separately in the top-down and bottom-up directions (Fig. 5).

EPS stimuli showed overall lower coupling strength than natural stimuli in both directions for both PACs and AACs (Fig. 5). Among PACs more specifically (Fig. 5*A*,*B*), natural stimuli showed higher coupling strength in the top-down direction for select frequency pairs in the low-frequency space as well as among low-frequency-to-high-gamma couplings (Fig. 5*A*). In the bottom-up direction, coupling strength for natural stimuli was enhanced among low-frequency-to-low- and high-gamma couplings (Fig. 5*B*). In the bottom-up direction, there were also select coupling values in the high-gamma frequency target amplitude space for which coupling was stronger in EPS stimuli.

Among AACs, coupling strength was increased for natural compared with EPS stimuli across the entire frequency spectrum in both directions (Fig. 5*E*,*F*). In particular, theta-gamma amplitude–amplitude interactions were significantly lower for EPS stimuli in both directions. Altogether, coupling strength is enhanced for natural stimuli over envelope-preserved but spectrally flat synthetic stimuli (i.e., EPS) in both directions and across the entire frequency spectrum.

SPS stimuli also showed overall lower coupling strength compared with natural stimuli. Among PACs, SPS stimuli showed lower coupling strength for select low-to-low- and low-to- high-frequency couplings in both directions (Fig. 5*C*,*D*). In the top-down direction, SPS stimuli showed higher coupling strength among gamma-gamma interactions (Fig. 5*D*). SPS and natural stimuli displayed similar differences among AACs (Fig. 5*G*,*H*). Altogether, coupling strength was mostly increased for natural stimuli compared with SPS stimuli across the entire frequency spectrum in both directions.

Comparing SPS and EPS stimuli directly (Extended Data Fig. 5-1), coupling strength was largely reduced for EPS stimuli across the frequency spectrum in both directions among PACs (Extended Data Fig. 5-1*A*,*B*). This was also true among AACs (Extended Data Fig. 5-1*C*,*D*), although there were fewer differences here than among PACs, especially in the high-frequency space.

Overall, top-down and bottom-up cross-frequency coupling signatures were preserved among synthetic stimuli, albeit at overall reduced coupling strength.

## Discussion

We found distinct cross-frequency signatures for top-down and bottom-up information processing in auditory cortex. These patterns were similar across phase–amplitude and amplitude–amplitude couplings, and across stimulus types (albeit at lower coupling strength in synthetic spectrum-flat stimuli). More specifically, we observed dominant top-down coupling among low-to-low- and low-to-high-gamma frequency interactions, and dominant bottom-up coupling among low-to-low-gamma frequency interactions. These patterns were largely preserved across coupling types (PAC and AAC) and across stimulus types (natural and synthetic stimuli), and mostly driven by interactions between A1 and higher-order sectors. Differences in coupling strength across the auditory hierarchy are overall an order of magnitude smaller among AACs than among PACs. Although the observed effects are subtle, they are significant and highly preserved. Altogether, our results suggest that these cross-frequency signatures are a general hallmark of bottom-up and top-down information processing in auditory cortex.

The top-down coupling profile we observed is in accordance with previous results, but we also observed predominant low-frequency-to-low-gamma coupling in the bottom-up direction. Previous studies found that feedforward (or bottom-up) influences were mediated by gamma frequency range rhythms (Bosman et al., 2012; Fontolan et al., 2014; Bastos et al., 2015; Michalareas et al., 2016). However, across each of these studies, there were examples of a broader frequency range being used for bottom-up information transmission. Fontolan et al. (2014) reported higher bottom-up coupling of delta (1–3 Hz) frequency phase in left A1 to high-gamma (80–100 Hz) amplitude in left association auditory cortex using human depth recordings. Bastos et al. (2015) found that theta channels were being used in addition to gamma-frequency channels in feedforward information transmission among primate visual areas. Finally, Michalareas et al. (2016) did not observe stronger alpha–beta frequency range feedback (top-down) modulation in 8 of 21 primate visual cortex area pairs (with 2 pairs showing the opposite pattern), and no stronger gamma frequency range feedforward (bottom-up) modulation in 5 of 21 pairs. We observed predominant bottom-up coupling between low-frequency (theta, alpha, beta) phase (or amplitude) and low-gamma amplitude among phase–amplitude couplings (and amplitude–amplitude couplings, respectively). Differences in the exact frequency ranges reported may be due to regional (visual vs auditory cortex) variation (Canolty et al., 2006; Tort et al., 2009) and species-related variation. Overall, our findings agree with accounts of predominant low-to-high (cross-) frequency interactions top-down. But together with the exceptions above, our findings suggest that low-to-high (cross-) frequency interactions may also play a role in bottom-up processing, pointing to a picture of top-down and bottom-up processing that is more complex than the previously hypothesized “beta-down versus gamma-up.”

Bottom-up PAC can be relevant to stimulus encoding. For example, an oscillation-based theory of speech encoding (Giraud and Poeppel, 2012; Hyafil et al., 2015a) predicts that cortical theta oscillations track the syllabic rhythm of speech and, in turn, reset the spiking of neurons at a gamma frequency level through theta-gamma PAC. This ensures that neural excitability is optimally aligned with incoming speech. Our finding of dominant low-frequency phase to gamma amplitude coupling bottom-up fits within a framework that requires theta–gamma coupling for accurate speech encoding (Hyafil et al., 2015a). Cross-regional theta–gamma PAC as we observed here across A1 and higher-order sectors could be responsible for coordinating information processing along the auditory cortical hierarchy. Our observation that these patterns are consistent across stimulus classes implies that this may be a generalized mechanism of auditory information processing that is co-opted during speech processing. However, our finding of enhanced low-to-high-frequency phase–amplitude coupling in the top-down direction shows that these couplings also mediate top-down influences on information processing.

Coupling in the top-down direction has been of particular interest in theories of the effect of attention on auditory information processing. Attention is thought to sample stimuli rhythmically, fluctuating at a 4–10 Hz rhythm (Landau and Fries, 2012), and was shown to enhance the processing of degraded speech in the anterior and posterior STS (Wild et al., 2012). Frontal top-down signals in the delta and theta frequency range have been shown to modulate low-frequency oscillations in human auditory cortex, increasing their coupling to continuous speech (Park et al., 2015). The same delta and theta frequency bands have been shown to be more strongly coupled to the gamma-range burst rate within monkey A1 during periods of task entrainment (Lakatos et al., 2016). In visual cortex, delta frequency oscillations have been shown to entrain with the rhythm of stimuli in the attended stream (Lakatos et al., 2008; Schroeder et al., 2010), and top-down beta-band modulation was found to be enhanced with attention (Bastos et al., 2015). Increased power in low-frequency range oscillations may also, in turn, enhance bottom-up rhythms through top-down (cross-frequency) coupling (Lee et al., 2013; Bressler and Richter, 2015); indeed, top-down beta-band activity was found to be maximally correlated with bottom-up gamma-band activity when top-down preceded bottom-up activity (Richter et al., 2017). In addition, top-down couplings may also reflect the modulatory activity of predictive processes on auditory/speech perception and processing (Poeppel et al., 2008; Wild et al., 2010 ; Davis et al., 2011; Arnal and Giraud, 2012; Gagnepain et al., 2012; Chao et al., 2018). Our findings of dominant low-to-low- and low-to-high-frequency coupling top-down are consistent with these accounts, and could well serve to enhance the engagement of the animal in the task (Park et al., 2015; Lakatos et al., 2016). In addition, the top-down coupling signal we observed may also be carrying predictive information, being consistent with frequency ranges that have been shown to carry prediction update signals (Chao et al., 2018). We cannot dissociate between attentive or predictive signals here, however.

We found PAC and AAC to offer similar accounts of information processing. This could be due to a high degree of shared informational content between the two measures; it remains unclear how these measures differ, and how AAC could function mechanistically in the brain. To the degree that PAC and AAC offer complementary, nonoverlapping information, it is possible that top-down influences are exerted more broadly through various CFC types, including both PAC and AAC, and others such as phase synchronization (Fries, 2005).

We observed the same pattern of results in synthetic stimuli. The overall lower coupling strength we observed in synthetic stimuli could be a consequence of the coding differences that were previously observed between synthetic and intact stimuli (Fukushima et al., 2014), or they could be driving these coding differences. Lower coupling strength in synthetic stimuli could also be a result of the modulatory effect of attention being lower for synthetic compared with natural stimuli, perhaps because animals pay less attention to unnatural, ecologically irrelevant stimuli. The observation of higher coupling strength in spectrally rich (SPS) over spectrally poor (EPS) stimuli, envelope-intact stimuli suggest enhanced encoding and processing of information-rich stimuli. This is also consistent with our previous results, which showed that information about spectrally rich stimuli was better maintained than information about spectrally flat stimuli, at least across the first two sectors (S1, S2) of the auditory hierarchy. In the highest-order auditory sector (S4), decoding performance showed differences across a broader frequency range among VOCs and EPSs than among VOCs and SPSs.

The fact that we find the same coupling patterns across natural and synthetic stimuli points to the existence of conserved cross-frequency signatures of bottom-up and top-down information processing in auditory cortex. These findings have important implications for theories of neural processing such as predictive coding. In theoretical implementations of predictive coding, the presence of nonlinearities in going from predictions to prediction errors may induce cross-frequency interactions in the top-down direction. These should be less prevalent if not absent in the bottom-up direction as prediction errors are thought to accumulate linearly. Our finding of predominant low-frequency to low-gamma cross-frequency coupling bottom-up suggests that nonlinearities may also play a role in bottom-up processing, allowing the modulation of gamma power in higher-order areas by the phase of low-frequency rhythms in lower-order areas, for example. Overall though, our finding of widespread predominant cross-frequency interactions in the top-down direction is in agreement with the predictive coding framework.

Many of the theoretical propositions for spectral asymmetries in forward and backwards message passing in predictive coding do not consider the role of attention, however. In predictive coding, attention is mediated by optimizing the precision of ascending prediction errors in a context-sensitive fashion. Physiologically, this corresponds to changing the gain of lower levels (usually considered to involve fast-spiking inhibitory interneurons and some form of coherence or synchronous gain). Crucially, the effect of attention adds an extra level of nonlinearity that could be mediated by backward connections, and, implicitly, cross-frequency coupling.

We used a method based on the canonical correlation framework that allowed us to estimate directed cross-frequency coupling strength in a computationally efficient framework. Many previous studies and methods to estimate cross-frequency coupling do not obtain directional estimates (Bruns and Eckhorn, 2004; Canolty et al., 2006; Cohen, 2008; Tort et al., 2010; Voytek et al., 2013; van Wijk et al., 2015), and thus do not allow for feedfoward and feedback components to be teased apart. Studies that do take direction into account (Bastos et al., 2012, 2015; Fontolan et al., 2014; Michalareas et al., 2016) use separate measures to look at coupling strength (e.g., through coherence) and directional information flow (e.g., through Granger causality), and do not take cross-frequency interactions into account. The dynamic causal modeling (DCM) framework (Friston et al., 2003, 2017; Chen et al., 2008) does allow for the computation of directed coupling across frequencies (Furl et al., 2014). Practically however, to enhance computational efficiency, current implementations of DCM reduce the dimensionality of the data by applying a singular value decomposition to the data from the source region *X*, and then projecting *Y* into this reduced space to compute coupling estimates (Chen et al., 2008). The variance preserving dimensions in the source and target space are not necessarily the dimensions that maximize correlations between source and target space, in which case interactions may be missed. In contrast, the canonical correlation framework used here has the advantage of finding low-dimensional representations that maximize coupling between the source (*X*) and target (*Y*) data. A similar approach has been used to obtain estimates of cross-frequency interactions in MEG data (Sato et al., 2010), however, without the directional Granger causal component included here.

In summary, we revealed distinct cross-frequency signatures of top-down and bottom-up information processing. These signatures are largely preserved across coupling types and stimulus types, suggesting that they are a general hallmark of information processing in auditory cortex. Our finding of prominent low-to-high-frequency coupling top-down extends the current view of low-to-high-frequency interactions in auditory cortex, which primarily emphasized their involvement in bottom-up processes. We used a method that allows for the computationally efficient estimation of directed cross-frequency interactions. Altogether, this extends our understanding of how information is propagated up and down the cortical hierarchy, with significant implications for theories of neural information processing such predictive coding.

## Synthesis

Reviewing Editor: Leonard Maler, University of Ottawa Decisions are customarily a result of the Reviewing Editor and the peer reviewers coming together and discussing their recommendations until a consensus is reached. When revisions are invited, a fact-based synthesis statement explaining their decision and outlining what is needed to prepare a revision will be listed below. The following reviewer(s) agreed to reveal their identity: Karl Friston.

The editor and reviewers all agreed that this Ms reports on very interesting research. The motivation behind this research, the experimental design and the resulting data set is very impressive. However, there are some flaws in the data analysis and its interpretation that are not convincing and that have to be corrected before this Ms is suitable for publication. The final version of the Ms should be shorter and use simpler and more appropriate analyses and statistics.

The reviewers have been very explicit in the required changes and so I have included their detailed comments below with only minor ediging.

Major points 

Conceptual: the way that you set up your hypotheses (and interpret your results) in relation to predictive coding could be improved. The predictions based upon predictive coding are not simply that bottom-up signals used high frequencies and top-down use low frequencies. Rather, the predictions are based upon the form of message passing implied by predictive coding. There are two aspects to this: first, the forward messages are passed from prediction error to expectation units so that expectation units accumulate linear mixtures of prediction errors. This means that forward influences can be cast as a low pass filter. In other words, high frequencies in a lower area will be attenuated relative to low frequencies, when expressed at higher levels. This can be contrasted with backward connections that mediate nonlinear predictions based upon expectations at higher levels. The implicit nonlinearity should induce cross frequency coupling - such that low frequency amplitude fluctuations in the higher area correlated with the amplitude of high frequencies in the lower area. This appears to be largely consistent with what you have observed. The key observation here is that the asymmetry between forward and backward connections lies in the asymmetry between linear (forward) and non-linear (backward) connections. This means that (non-linear) cross frequency coupling should be greater in the backward connections, compared to the forward connections; irrespective of the particular frequencies involved. Perhaps you could unpack this in a bit more detail (I think these points were covered in the Friston 2008 paper you cite)? 

Cross frequency coupling analysis: I thought your use of canonical correlation analysis was clever and was nicely motivated by appeal to Granger causality. However, you need to describe more clearly the nature of your linear model of coupling. The equations suggest that you used two time lags for the CCA. However, it was not clear what these lags were (e.g., 200 ms?) Second, if you used two time lags, why did you not use one time or 16 lags? I ask this because the CCA is based upon a linear model of between frequency coupling. The introduction of time lags makes this into a convolution or autoregressive model. So, how did you optimise the number of time lags and how did you optimise the duration of the lags? Mathematically speaking, the model of cross frequency coupling is the same as that used in dynamic causal modelling for induced responses; however, DCM does not make any ad hoc assumptions about instantaneous or unidirectional coupling in implicit in your CCA. You should take a look at Chen et al and (2008): 

https://www.ncbi.nlm.nih.gov/pubmed/18485744 

These authors used DCM for cross frequency coupling to address a similar issue in the visual system; namely, the amplitude to amplitude coupling across different frequencies. It would be interesting to see how you motivate your approach in light of this sort of (DCM) analysis. 

Strategically: your paper is a bit too long for its own good. I think you can shorten it in several ways. First, you can describe your CCA analysis more succinctly. Perhaps with something like: 

“To characterise amplitude (and phase)-amplitude coupling, we extracted the amplitude envelopes over sequences using a Hilbert transform and then used canonical correlation analysis to parameterise the cross frequency coupling under a general linear model. Crucially, the fluctuations in amplitude in the target sector were predicted on the basis of time lagged amplitude fluctuations in the source sector. Furthermore, the target and source time series were adjusted to remove the effect of predicted fluctuations from the target region. In other words, by appealing to the notion of Granger causality, a significant mapping between the source and the target - that cannot be explained by the history of the target - is evidence of (cross frequency) coupling.” 

Please remove your analysis of phase-amplitude coupling over time. Although you did not specify the window length for this analysis, Figure 7 suggests that this was in the order of several hundred milliseconds. You cannot assess cross frequency coupling with theta at a time resolution less than the Nyquist limit. This renders the interpretation of the results in Figure 7 obscure. Even if you used some analysis that was based upon suitably long time windows, I am not sure what your hypothesis was here. In short, a lot of the results towards the end of your results section were redundant because there was too much information of a purely descriptive sort. You should try to substantially reduce the length of the paper by reporting just those analyses that speak to your hypotheses. This may ensure people will remember your results and your work has more impact. 

Statistics: The method you have chosen to correct for multiple comparisons is not valid. You have applied FDR to each frequency-frequency bin. This is not appropriate in the context of continuous coupling matrices of this sort. You could have applied FDR to the Maxim or other topological features (in a similar way to the topological inference based upon your cluster randomisation approach). You can read more about this in: 

https://www.ncbi.nlm.nih.gov/pubmed/18603449 

This means, one cannot be sure whether any of your differences are significant. Furthermore, it is not clear what the degrees of freedom were in your t-test. In other words, when you performed your t-tests on the difference between forward and backward coupling coefficients, where these tests over subjects, over multiple sessions or multiple time windows? What were the degrees of freedom of these tests? You may have described this in supporting material but I could not find this information in the main text. My advice would be to smooth your frequency-frequency coupling measures (and differences) and then apply standard topological inference for smooth data: see for example: 

https://www.ncbi.nlm.nih.gov/pubmed/19944173 

Alternatively, you can reduce the resolution of your frequency-frequency coupling matrices to render a Bonferroni correction appropriate (e.g., using 8 Hz frequency bins). I think you will then get a clear picture of what is significant and what is not. 

Discussion: I enjoyed your discussion, which was useful and insightful. I think you can make more of the attentional influences on top-down coupling. Perhaps with something like: 

“Note that much of the theoretical propositions for spectral asymmetries in forward and backward message passing in predictive coding do not consider the key role of attention. In predictive coding, attention is mediated by optimising the precision of ascending prediction errors in a context sensitive fashion. Physiologically, this corresponds to changing the gain of lower levels (usually considered to involve fast spiking inhibitory interneurons and some form of coherence or synchronous gain). Crucially, this adds an extra level of nonlinearity that could be mediated by backward connections and, implicitly, cross frequency coupling.” 

There were a couple of phrases I think you can remove from the discussion. For example, “both mechanisms may be at play in regulating information processing in the two directions”. Could you suppress these empty assertions - the promise of your work is that you have a particular hypothesis about recurrent message passing in cortical hierarchies that must involve cross frequency coupling if this message passing is nonlinear. 

I would also be careful about asserting that “we also found more prominent involvement of low frequencies in bottom up information processing”. Did you find evidence for this - or did you find evidence that the differences between bottom-up and top-down involved low frequencies? 

Minor points 

On page 7, please replace “multivarite” with “multivariate” 

I would be tempted to remove equations (1) and (2). You would need to explain all the variables in detail; especially the rather odd Y_t1,t2 variable in the first equality (which I assume is a time lagged variable). I would just assume that readers will know what canonical correlation (i.e. covariates) analysis is (or can go look it up on Wikipedia). 

On page 8 and elsewhere, you refer to ‘singular value’. Did you mean the ‘canonical values’ associated with the canonical correlations? 

On page 24, could you replace ‘to high frequency coupling’ with ‘to high-frequency top-down coupling’, halfway down the page. 

- the authors use canonical correlation analysis to derive PAC and AAC measures. This seems to be a relatively recent application of canonical correlation analysis. I found that Soto et al. (2016) have used it for MEG. 

- I think it would help the reader interested in the methods to explicitly describe the dimensionality of P, A, X, etc ... 

- although it does not seem to be one of the main results of the paper, the authors state that spectrum preserving sounds are more similar to natural vocalization sounds than the envelope preserving versions. However, to make such a claim one would need to numerically compare the differences, which is not done in the manuscript. 

- I think that the methods section refers to STP and STG without defining them first.

## Author Response

Dear Dr. Maler,

Thanks for conveying the news and the summary of responses. We also appreciate the time and care the reviewers took to assess our work. We have modified the manuscript based on the feedback, and have included detailed responses below. Altogether the reviewers' comments have helped us in making the revised version of the manuscript tighter and more approachable.

Best,

--------------------------------------------- Synthesis of Reviews:

Computational Neuroscience Model Code Accessibility Comments for Author

(Required):

not applicable

Significance Statement Comments for Author (Required): OK

Comments on the Visual Abstract for Author (Required): N/A

Synthesis Statement for Author (Required):

The editor and reviewers all agreed that this Ms reports on very interesting research. The motivation behind this research, the experimental design and the resulting data set is very impressive. However, there are some flaws in the data analysis and its interpretation that are not convincing and that have to be corrected before this Ms is suitable for publication. The final version of the Ms should be shorter and use simpler and more appropriate analyses and statistics.

The reviewers have been very explicit in the required changes and so I have included their detailed comments below with only minor editing.

Altogether, we have revised our manuscript to be shorter (by reducing the figure count and more succinctly summarising our results, as well as by compressing our discussion), have revised our figures to reflect appropriate statistical analyses, and

made our analysis method more approachable by incorporating more detail in some areas and leaving it out in others (as suggested) and by relating it to other approaches.

Major points

Conceptual: the way that you set up your hypotheses (and interpret your results) in relation to predictive coding could be improved. The predictions based upon predictive coding are not simply that bottom-up signals used high frequencies and top-down use low frequencies. Rather, the predictions are based upon the form of message passing implied by predictive coding. There are two aspects to this: first, the forward messages are passed from prediction error to expectation units so that expectation units accumulate linear mixtures of prediction errors. This means that forward influences can be cast as a low pass filter. In other words, high frequencies in a lower area will be attenuated relative to low frequencies, when expressed at higher levels. This can be contrasted with backward connections that mediate nonlinear predictions based upon expectations at higher levels. The implicit nonlinearity should induce cross frequency coupling - such that low frequency amplitude fluctuations in the higher area correlated with the amplitude of high frequencies in the lower area. This appears to be largely consistent with what you have observed. The key observation here is that the asymmetry between forward and backward connections lies in the asymmetry between linear (forward) and non-linear (backward) connections. This means that (non-linear) cross frequency coupling should be greater in the backward connections, compared to the forward connections; irrespective of the particular frequencies involved. Perhaps you could unpack this in a bit more detail (I think these points were covered in the Friston 2008 paper you cite)?

Thanks for these remarks. We acknowledge that we could have been more clear and have updated the way we lay out our hypotheses and present our results. We made changes to the abstract, significance statement, introduction and discussion to reflect these points.

Abstract:

“The theory suggests there are differences in message passing upwards and downwards the cortical hierarchy. These differences result from the linear feedforward of prediction errors, and the nonlinear feedback of predictions. This implies that cross- frequency interactions should predominate top-down. But it remains unknown whether these differences are expressed in cross-frequency interactions in the brain.” (...) “Our findings suggest the modulatory effect of low frequencies on $\gamma$-rhythms in distant regions is important for bi-directional information transfer. The finding of low frequency-to-$low\, \gamma$ interactions in the bottom-up direction suggest non- linearities may also play a role in feedforward message passing. Altogether, the patterns of cross-frequency interaction we observed across the auditory hierarchy are largely consistent with the predictive coding framework.”

Significance statement:

“Our findings reveal cross-frequency interactions are predominant in the top-down direction, with important implications for theories of information processing in the brain such as predictive coding. Our findings thereby extend the view of cross-frequency interactions in auditory cortex, suggesting they also play a prominent role in top-down processing.”

Introduction:

“Neural implementations of predictive coding suggest that predictions are formed in higher-order areas through a (linear) accumulation of prediction errors. The accumulation of prediction errors in higher-order areas results in the attenuation of high frequencies, thus feedforward connections can be cast as a low-pass or Bayesian filter. Conversely, prediction errors arising in lower-order areas are a non-linear function of predictions. This means high frequencies can be created in lower-order areas due to the inherent nonlinearity. Hence, predictive coding implies that lower-order areas (where prediction errors arise) should display relatively higher frequencies (e.g. $

\gamma$) than higher-order areas (where predictions are formed) ~\cite{Bastos:

2012bd, Friston:2008cf}.” (...)

It remains unknown whether this prediction is expressed in cross-frequency interactions in the brain. Here we probe this prediction by examining differences in cross-frequency coupling strength in the bottom-up and top-down direction across the frequency spectrum. \textcolor{blue}{Based on the predictive coding framework

~\cite{Bastos:2012bd, Friston:2008cf}, we would expect there to be an asymmetry between bottom-up and top-down interactions. This asymmetry arises due to the linear effect of prediction errors on predictions in the bottom-up direction, and the nonlinear effect of predictions on prediction erros in the top-down direction. Thus, we would expect nonlinear, \textit{cross}-frequency interactions to predominate in the top-down direction.

Discussion:

“We found distinct cross-frequency signatures for top-down and bottom-up information processing in auditory cortex. These patterns were similar across phase- amplitude and amplitude-amplitude couplings, and across stimulus types (albeit at lower coupling strength in synthetic spectrum-flat stimuli). More specifically, we observed dominant top-down coupling among low-to-low and low-to-high $\gamma$ frequency interactions, and dominant bottom-up coupling among low-to-low $

\gamma$ frequency interactions. These patterns were largely preserved across coupling types (PAC and AAC) and across stimulus types (natural and synthetic stimuli). Altogether, our results suggest these cross-frequency signatures are a general hallmark of bottom-up and top-down information processing in auditory cortex.”

(...)

“ O v e r a l l , o u r fi n d i n g s a g re e w i t h a c c o u n t s o f p re d o m i n a n t l o w - t o - h i g h (cross-)frequency interactions top-down. But together with the exceptions above, our findings suggest low-to-high (cross-)frequency interactions may also play a role in bottom-up processing, pointing to a picture of top-down and bottom-up processing

that is more complex than the previously hypothesized ‘$\beta$-down vs. $\gamma$- up’ ~\cite{Bastos:2012bd,Fontolan:2014bx}.”

(...)

“In summary, we revealed distinct cross-frequency signatures of top-down and bottom-up information processing. These signatures are largely preserved across coupling types and stimulus types, suggesting they are a general hallmark of information processing in auditory cortex. Our finding of prominent low-to-high frequency coupling top-down extends the current view of low-to-high frequency interactions in auditory cortex, which primarily emphasized their involvement in bottom-up processes. We employed a method that allows for the computationally eﬃcient estimation of directed cross-frequency interactions. Altogether, this extends our understanding of how information is propagated up and down the cortical hierarchy, with significant implications for theories of neural information processing such predictive coding.”

Cross frequency coupling analysis: I thought your use of canonical correlation analysis was clever and was nicely motivated by appeal to Granger causality. However, you need to describe more clearly the nature of your linear model of coupling. The equations suggest that you used two time lags for the CCA. However, it was not clear what these lags were (e.g., 200 ms?) Second, if you used two time lags, why did you not use one time or 16 lags? I ask this because the CCA is based upon a linear model of between frequency coupling. The introduction of time lags makes this into a convolution or autoregressive model. So, how did you optimise the number of time lags and how did you optimise the duration of the lags? Mathematically speaking, the model of cross frequency coupling is the same as that used in dynamic causal modelling for induced responses; however, DCM does not make any ad hoc assumptions about instantaneous or unidirectional coupling in implicit in your CCA. You should take a look at Chen et al and (2008): https://www.ncbi.nlm.nih.gov/pubmed/18485744 ;

These authors used DCM for cross frequency coupling to address a similar issue in the visual system; namely, the amplitude to amplitude coupling across different frequencies. It would be interesting to see how you motivate your approach in light of this sort of (DCM) analysis.

Thanks for these questions. To be more clear, we used two time lags for the autocorrelation step of the analyses where we removed the effect of predicted fluctuations from the target region. We did not systematically explore different time lags, but we used a low number of lags for the purpose of computational eﬃciency. We found that it did not affect our results whether we used 1 or 2 lags, however. We updated the methods section to clarify these points:

“We carried out our analyses using a Granger Causal framework. Therefore, we first carried out CCA between amplitude in the target region and time lagged versions of the amplitude, obtaining an estimate of the the coupling strength $\widehat{Y}$ that could

be inferred purely from coupling of the signal at the target electrode onto itself. For computational reasons, we used two time lags to estimate the target signal (we found that using one time lag did not affect our results). More explicitly, we transformed current and lagged signal into the frequency domain as described above and computed the canonical correlation between the lagged components and the current signal in the target. We used the first canonical value only to obtain $\widehat{Y}$ (results did not differ from those obtained from including all entries). We then obtained the residual $\widetilde{Y}$ from this analysis, and used this residual when we examined the interactions between regions. Subsequent analysis was performed using the residuals $\widetilde{Y}$ from this regression as the signal for the target region.”

When computing coupling estimates within the CCA framework, we estimated instantaneous coupling between source and target region in 200ms windows. The larger window size arises due to time-frequency tradeoffs. As processing delays across the auditory hierarchy (on the order of 10s of msecs) are much smaller than this window size, they are captured in the frequency profile and should not adversely affect coupling estimates (which are based on much larger durations). We updated the methods section in the following manner:

“We then computed phase-amplitude (PAC) and amplitude-amplitude (AAC) cross- frequency couplings across the four sectors (Fig. \ref{fig:Figure 1}), by regressing the phase or amplitude component of the signal from the source sector, respectively, on the amplitude component of the signal in the target sector for every channel. More specifically, we calculated instantaneous coupling in 200ms windows between source and target region for each electrode pair. PAC and AAC was calculated in the CCA framework across all time windows and trials altogether, simultaneously across all frequency bands.”

As to how our method compares to other approaches to estimating cross-frequency coupling, in particular DCM, we have actually employed DCM in a previous paper (Furl et. al. 2015 Cerebral Cortex) and realized that current implementations (Chen et al

2008, p. 1302) apply dimensionality reduction (SVD) to the source and target signal separately, or project the target signal into the space spanned by the directions of highest variance derived from the source signal alone. The problem with this approach is that the directions of highest variance in source and target signal may not necessarily be the directions that maximise coupling between the two signals. In contrast, in CCA we obtain the directions of highest variance that also maximise coupling, and can simultaneously estimate same- and cross-frequency coupling across all frequencies altogether. Moreover, in contrast to most previous approaches, we obtain directional estimates of cross-frequency coupling with our approach. We have included this paragraph in the discussion to clarify how our method relates to previous approaches:

“We employed a method based on the canonical correlation framework that allowed us to estimate directed cross-frequency coupling strength in a computationally eﬃcient framework. Many previous studies and methods to estimate cross-frequency coupling do not obtain directional estimates ~\cite{Cohen:2008jm,Canolty:2006jm,Bruns:

2004kd,Tort:2010ge,Voytek:2013fma,vanWijk:2015hv}, and thus do not allow for feedfoward and feedback components to be teased apart. Studies that do take d i re c t i o n i n t o a c c o u n t ~ \ c i t e { B a s t o s : 2 0 1 2 b d , M i c h a l a re a s : 2 0 1 6 c g , B a s t o s :

2015eu,Fontolan:2014bx} employ separate measures to look at coupling strength (e.g. through coherence) and directional information flow (e.g. through Granger causality), and do not take \textit{cross}-frequency interactions into account. The dynamic causal modelling (DCM) framework ~\cite{Friston:2017gy,Friston:2003cv,Chen:2008kl} does allow for the computation of directed coupling across frequencies ~\cite{Furl:2014if}. Practically however, to enhance computational eﬃciency, current implementations of DCM reduce the dimensionality of the data by applying a singular value decomposition to the data from the source region $X$, and then projecting $Y$ into this reduced space to compute coupling estimates ~\cite{Chen:2008kl}. The variance preserving dimensions in the source and target space are not necessarily the dimensions that maximize correlations between source and target space, in which case interactions may be missed. In contrast, the canonical correlation framework employed here has the advantage that it finds low dimensional representations which maximize coupling between the source ($X$) and target ($Y$) data. A similar approach has been used recently to obtain estimates of cross-frequency interactions in MEG data ~\cite{Sato:

2010gu}, however without the directional Granger-causal component included here.”

Strategically: your paper is a bit too long for its own good. I think you can shorten it in several ways. First, you can describe your CCA analysis more succinctly. Perhaps with something like:

“To characterise amplitude (and phase)-amplitude coupling, we extracted the amplitude envelopes over sequences using a Hilbert transform and then used canonical correlation analysis to parameterise the cross frequency coupling under a general linear model. Crucially, the fluctuations in amplitude in the target sector were predicted on the basis of time lagged amplitude fluctuations in the source sector. Furthermore, the target and source time series were adjusted to remove the effect of predicted fluctuations from the target region. In other words, by appealing to the notion of Granger causality, a significant mapping between the source and the target - that cannot be explained by the history of the target - is evidence of (cross frequency) coupling.”

We have revised this part of our results to reflect this suggestion.

“We wanted to examine cross frequency interactions across the auditory hierarchy. To characterise phase-amplitude and amplitude-amplitude coupling, we first decomposed the signal into its spectral component using the short-time fast Fourier transform and obtained phase and amplitude components (Fig. \ref{fig:Figure 1}). We then used canonical correlation analysis (CCA) to parameterise the cross-frequency coupling under a general linear model (\nameref{section:Methods}). The fluctuations in amplitude in the target sector were predicted based on instantaneous amplitude fluctuations in the source sector within 200ms windows. Furthermore, the target and

source time series were adjusted to remove the effect of predicted fluctuations from the target region. In other words, by appealing to the notion of Granger causality, a significant mapping between the source and the target - that cannot be explained by the history of the target - is evidence of (cross-frequency) coupling.”

Please remove your analysis of phase-amplitude coupling over time. Although you did not specify the window length for this analysis, Figure 7 suggests that this was in the order of several hundred milliseconds. You cannot assess cross frequency coupling with theta at a time resolution less than the Nyquist limit. This renders the interpretation of the results in Figure 7 obscure. Even if you used some analysis that was based upon suitably long time windows, I am not sure what your hypothesis was here. In short, a lot of the results towards the end of your results section were redundant because there was too much information of a purely descriptive sort. You should try to substantially reduce the length of the paper by reporting just those analyses that speak to your hypotheses. This may ensure people will remember your results and your work has more impact.

We have removed the analysis of phase-amplitude coupling over time upon your suggestion. We have also compressed our results from two separate figures into one (now Figure 5) and expressed our results more succinctly.

Statistics: The method you have chosen to correct for multiple comparisons is not valid. You have applied FDR to each frequency-frequency bin. This is not appropriate in the context of continuous coupling matrices of this sort. You could have applied FDR to the Maxim or other topological features (in a similar way to the topological inference based upon your cluster randomisation approach). You can read more about this in: https://www.ncbi.nlm.nih.gov/pubmed/18603449 ;

This means, one cannot be sure whether any of your differences are significant. Furthermore, it is not clear what the degrees of freedom were in your t-test. In other words, when you performed your t-tests on the difference between forward and backward coupling coeﬃcients, where these tests over subjects, over multiple sessions or multiple time windows? What were the degrees of freedom of these tests? You may have described this in supporting material but I could not find this information in the main text. My advice would be to smooth your frequency-frequency coupling measures (and differences) and then apply standard topological inference for smooth data: see for example:

https://www.ncbi.nlm.nih.gov/pubmed/19944173 ;

Alternatively, you can reduce the resolution of your frequency-frequency coupling matrices to render a Bonferroni correction appropriate (e.g., using 8 Hz frequency bins). I think you will then get a clear picture of what is significant and what is not.

We have re-run our statistical analyses and updated all of the figures to reflect significant interactions based on the non-parametric cluster-correction method (Maris

& Oostenveld, 2007; Nichols & Holmes, 2001). As an alternative to the approaches suggested above, this approach has been previously used to characterise the significance of cross-frequency interactions (Fontolan et. al. 2014, Nat. Comms.). We had included figures of the main results using this method in the supplementary figures before, but we have now updated all figures to reflect this approach. We conducted our t-tests across all channel pairs, cross regional pairs and animals altogether, unless otherwise indicated. This information had already been in the “statistical analysis” section of the results before, but we have updated that section to also report degrees of freedom. We have updated the methods section to reflect these changes:

“Paired t-tests were performed to establish statistical significance of the difference between top-down and bottom-up coupling ($\Delta P$; Fig. \ref{fig:Figure 2}, Fig.

\ref{fig:Figure 3}, Fig. \ref{fig:Figure 4}), as well as the differences between original and synthetic stimuli ($P^{TD}_{\,VOC\,vs\,Synthetic}$, $P^{BU}_{\,VOC\,vs\,Synthetic}$; Fig. \ref{fig:Figure 5}). This was done separately for each frequency pair, by testing whether the mean of the difference between top-down and bottom-up coupling (or the difference between original and synthetic stimuli in each direction) was significantly different from zero (null hypothesis). Lilliefors' test for normality was conducted for each frequency pair in order to ensure the normality assumption was satisfied, and histogram plots were inspected for select frequency pairs (Fig. \ref{fig:Figure S2},

\ref{fig:Figure S3}).\More specifically, in order to assess overall differences in coupling strength between the two directions (Fig. \ref{fig:Figure 2},Fig. \ref{fig:Figure 4}) and to assess differences in coupling strength between natural and synthetic stimuli (Fig. \ref{fig:Figure 5}), paired t-tests were conducted across all channel pairs, cross-regional pairs and animals altogether ($d.f.=9344$). In order to assess coupling differences for each regional pair separately (Fig. \ref{fig:Figure 3}), paired t-tests were conducted separately for each regional pair, across all channel pairs and animals altogether ($d.f.=1056$).\Significant phase-amplitude and amplitude-amplitude coupling (highlighted by black outlines throughout the manuscript) was based on corrected p-values using non- parametric permutation tests that generated null distributions of the maximum cluster size ~\cite{Maris:2007ir}. This procedure implicitly adjusts for searching over multiple frequencies. The null distribution was obtained by randomly reassigning electrodes to source or target region and recalculating differences in coupling strength for 100 repeats. Clusters of the 99th percentile (corresponding to p$\leq$0.01) were considered significant and are reported throughout the manuscript (marked by black outlines).”

Discussion: I enjoyed your discussion, which was useful and insightful. I think you can make more of the attentional influences on top-down coupling. Perhaps with something like:

“Note that much of the theoretical propositions for spectral asymmetries in forward and backward message passing in predictive coding do not consider the key role of attention. In predictive coding, attention is mediated by optimising the precision of ascending prediction errors in a context sensitive fashion. Physiologically, this corresponds to changing the gain of lower levels (usually considered to involve fast spiking inhibitory interneurons and some form of coherence or synchronous gain). Crucially, this adds an extra level of nonlinearity that could be mediated by backward connections and, implicitly, cross frequency coupling.”

Thanks for the suggestion, we have included a version of this statement in the discussion:

“Many of the theoretical propositions for spectral asymmetries in forward and backwards message passing in predictive coding do not consider the role of attention, however. In predictive coding, attention is mediated by optimising the precision of ascending prediction errors in a context-sensitive fashion. Physiologically, this corresponds to changing the gain of lower levels (usually considered to involve fast spiking inhibitory interneurons and some form of coherence or synchronous gain). Crucially, the effect of attention adds an extra level of nonlinearity that could be mediated by backward connections, and, implicitly, cross frequency coupling.”

There were a couple of phrases I think you can remove from the discussion. For example, “both mechanisms may be at play in regulating information processing in the two directions”. Could you suppress these empty assertions - the promise of your work is that you have a particular hypothesis about recurrent message passing in cortical hierarchies that must involve cross frequency coupling if this message passing is nonlinear.

We have removed that sentence and updated this part of the Discussion:

“The fact that we find the same coupling patterns across natural and synthetic stimuli points to the existence of conserved cross-frequency signatures of bottom-up and top- down information processing in auditory cortex. These findings have important implications for theories of neural processing such as predictive coding. In theoretical implementations of predictive coding, the presence of nonlinearities in going from predictions to prediction errors may induce cross-frequency interactions in the top- down direction. These should be less prevalent if not absent in the bottom-up direction as prediction errors are thought to accumulate linearly. Our finding of predominant low frequency to $low \gamma$ cross-frequency coupling bottom-up suggests nonlinearities may also play a role in bottom-up processing, allowing the modulation of

$\gamma$ power in higher-order areas by the phase of low frequency rhythms in lower order areas, for example. Overall though, our finding of widespread predominant cross- frequency interactions in the top-down direction is in agreement with the predictive coding framework.”

I would also be careful about asserting that “we also found more prominent involvement of low frequencies in bottom up information processing”. Did you find evidence for this - or did you find evidence that the differences between bottom-up and top-down involved low frequencies?

We have updated this part of the Discussion:

“The top-down coupling profile we observed is in accordance with previous results, but we also observed predominant low frequency to $\low \gamma$ coupling in the bottom-up direction. Previous studies found that feedforward (or bottom-up) influences were mediated by gamma frequency range rhythms ~\cite{Michalareas:2016cg, Bastos:2015eu, Fontolan:2014bx, Bosman:2012ej}.} However, across each of these studies, there were examples of a broader frequency range being employed for bottom-up information transmission. ~\cite{Fontolan:2014bx} reported higher bottom- up coupling of delta (1-3Hz) frequency phase in left A1 to high gamma (80-100Hz) amplitude in left association auditory cortex using human depth recordings.

~\cite{Bastos:2015eu} found that theta-channels were being used in addition to gamma-frequency channels in feedforward information transmission among primate visual areas. Finally, ~\cite{Michalareas:2016cg} did not observe stronger alpha-beta frequency range feedback (top-down) modulation in 8 out of 21 primate visual cortex area pairs (with 2 pairs showing the opposite pattern), and no stronger gamma frequency range feedforward (bottom-up) modulation in 5 out of 21 pairs. We observed predominant bottom-up coupling between low frequency ($\theta$,$\alpha$,$\beta$) phase (or amplitude) and low-$\gamma$ amplitude among phase-amplitude couplings (and amplitude-amplitude couplings,respectively). Differences in exact frequency ranges reported may be due to regional (visual versus auditory cortex) variation

~\cite{Tort:2009uc,Canolty:2006jm} and species-related variation. \textcolor{blue}

{ O v e r a l l , o u r fi n d i n g s a g re e w i t h a c c o u n t s o f p re d o m i n a n t l o w - t o - h i g h (cross-)frequency interactions top-down. But together with the exceptions above, our findings suggest low-to-high (cross-)frequency interactions may also play a role in bottom-up processing, pointing to a picture of top-down and bottom-up processing that is more complex than the previously hypothesized ‘$\beta$-down vs. $\gamma$- up’ ~\cite{Bastos:2012bd,Fontolan:2014bx}.”

Minor points

On page 7, please replace ‘multivarite’ with ‘multivariate’

I would be tempted to remove equations (1) and (2). You would need to explain all the variables in detail; especially the rather odd Y_t1,t2 variable in the first equality (which I assume is a time lagged variable). I would just assume that readers will know what canonical correlation (i.e. covariates) analysis is (or can go look it up on Wikipedia).

We have removed these equations upon your suggestion and kept our description in words. We have kept equations 3 and 4, however, as we believe they serve to enhance the reader's understanding of how couplings are calculated in the CCA framework.

On page 8 and elsewhere, you refer to “singular value”. Did you mean the “canonical values” associated with the canonical correlations?

Yes, we have replaced these throughout.

On page 24, could you replace “to high frequency coupling” with “to high-frequency top-down coupling”, halfway down the page.

“Our findings of dominant low-to-low and low-to-high frequency coupling top-down are consistent with these accounts, and could well serve to enhance the animals engagement in the task ~\cite{Lakatos:2016ih,Park:2015fo}.”

- the authors use canonical correlation analysis to derive PAC and AAC measures. This seems to be a relatively recent application of canonical correlation analysis. I found that Soto et al. (2016) have used it for MEG.

Thanks for this suggestion, we have included this in the Discussion:

“A similar approach has been used recently to obtain estimates of cross-frequency interactions in MEG data (cite), however without the directional Granger-causal component included here.”

- I think it would help the reader interested in the methods to explicitly describe the dimensionality of P, A, X, etc ...

Thanks for this suggestion, we have included the dimensionality of these variables in the text as well as in the equations:

“In the case of PAC, $X_{n\,x\,d1}$ contained the sine and cosine transformed phase component of the signal, while $Y_{n\,x\,d2}$ contained the squared and log- transformed amplitude component of the signal, with $n=10800$ (1200 trials * 9 windows) observations for VOC and SPS stimuli and $n=10773$ observations (1197 trials * 9 windows) for EPS stimuli, $d2=25$, and $d1=25$ or $d1=25*2$ for PAC and AAC, respectively (Eq \ref{eq:CANONCORR_Y3}).}\

After obtaining the canonical coeﬃcient matrices $B_{d2\,x\,d}$ and $A_{d1\,x\,d}$ within the CCA framework (Eq \ref{eq:CANONCORR_Y3}), one can obtain the coupling matrix $P_{d2\,x\,d1}$ by multiplying the $B$ and $A$ matrices with the canonical values $S$ of the correlation matrix (Eq \ref{eq:CANONCORR_Y4}). We used the first

10 directions ($d=10$) that captured most of the interaction; results did not differ from those obtained from including all entries. For AAC, the final cross-frequency coupling

matrix is $P$. For PAC, one combines the sine and cosine components of $P$ by computing their Euclidian norm, yielding the final coupling matrix $P^{*}_{d2\,x\,d1}$ with $d2=d1$.\\ ”

- although it does not seem to be one of the main results of the paper, the authors state that spectrum preserving sounds are more similar to natural vocalization sounds than the envelope preserving versions. However, to make such a claim one would need to numerically compare the differences, which is not done in the manuscript.

We have removed these claims from the results and discussion.

- I think that the methods section refers to STP and STG without defining them first. “The 96 sites on the supratemporal plane (STP) were grouped based on the characteristic frequency maps obtained from the high-gamma power of the evoked response to a set of pure-tone stimuli.”
